# Cognitive rehabilitation and mindfulness in multiple sclerosis (REMIND-MS): a study protocol for a randomised controlled trial

**DOI:** 10.1186/s12883-017-0979-y

**Published:** 2017-11-21

**Authors:** Ilse M. Nauta, Anne E. M. Speckens, Roy P. C. Kessels, Jeroen J. G. Geurts, Vincent de Groot, Bernard M. J. Uitdehaag, Luciano Fasotti, Brigit A. de Jong

**Affiliations:** 1 Department of Neurology, Amsterdam Neuroscience, MS Center Amsterdam, VU University Medical Center, PO Box 7057, 1007 MB Amsterdam, the Netherlands; 2 Department of Psychiatry, Radboud University Medical Center, PO Box 9101, 6500 HB Nijmegen, the Netherlands; 3 Donders Institute for Brain, Cognition and Behaviour, Radboud University, PO Box 9101, 6500 HB Nijmegen, the Netherlands; 4 Department of Medical Psychology, Radboud University Medical Center, PO Box 9101, 6500 HB Nijmegen, the Netherlands; 5 Department of Anatomy and Neurosciences, Amsterdam Neuroscience, MS Center Amsterdam, VU University Medical Center, PO Box 7057, 1007 MB Amsterdam, the Netherlands; 6 Department of Rehabilitation Medicine, MS Center Amsterdam, VU University Medical Center, PO Box 7057, 1007 MB Amsterdam, the Netherlands; 7 Klimmendaal Rehabilitation Center, PO Box 9044, 6800 CG Arnhem, the Netherlands

**Keywords:** Multiple sclerosis, Cognition, Cognitive rehabilitation therapy, Mindfulness-based cognitive therapy, Brain networks, Randomised controlled trial

## Abstract

**Background:**

Cognitive problems frequently occur in patients with multiple sclerosis (MS) and profoundly affect their quality of life. So far, the best cognitive treatment options for MS patients are a matter of debate. Therefore, this study aims to investigate the effectiveness of two promising non-pharmacological treatments: cognitive rehabilitation therapy (CRT) and mindfulness-based cognitive therapy (MBCT). Furthermore, this study aims to gain additional knowledge about the aetiology of cognitive problems among MS patients, since this may help to develop and guide effective cognitive treatments.

**Methods/design:**

In a dual-centre, single-blind randomised controlled trial (RCT), 120 MS patients will be randomised into one of three parallel groups: CRT, MBCT or enhanced treatment as usual (ETAU). Both CRT and MBCT consist of a structured 9-week program. ETAU consists of one appointment with an MS specialist nurse. Measurements will be performed at baseline, post-intervention and 6 months after the interventions. The primary outcome measure is the level of subjective cognitive complaints. Secondary outcome measures are objective cognitive function, functional brain network measures (using magnetoencephalography), psychological symptoms, well-being, quality of life and daily life functioning.

**Discussion:**

To our knowledge, this will be the first RCT that investigates the effect of MBCT on cognitive function among MS patients. In addition, studying the effect of CRT on cognitive function may provide direction to the contradictory evidence that is currently available. This study will also provide information on changes in functional brain networks in relation to cognitive function. To conclude, this study may help to understand and treat cognitive problems among MS patients.

**Trial registration:**

This trial was prospectively registered at the Dutch Trial Registration (number NTR6459, registered on 31 May 2017).

## Background

Multiple sclerosis (MS) is a chronic disease of the central nervous system, which leads to physical, neuropsychiatric and cognitive problems. Cognitive problems are commonly reported by MS patients, with prevalence rates of objective cognitive deficits varying between 43 and 70% [1]. The most frequently affected cognitive domains are information processing speed, memory, attention, visuospatial processing and executive function. These objective cognitive deficits (i.e. assessed with cognitive tests) only show a weak relation with the cognitive complaints reported by MS patients themselves [2, 3]. Despite this weak relation, subjectively experienced cognitive problems are arguably as important as objective cognitive deficits, since they may reflect the burden of cognitive problems in daily life.

The impact of cognitive problems on daily life can be extensive given the relatively young age of disease onset. Problems in social relations and work participation are likely to occur, consequently negatively affecting the quality of life of MS patients [1, 4]. This highlights the need for effective cognitive treatment options for MS patients. To develop and guide effective cognitive treatments, knowledge about the aetiology of objective and subjective cognitive problems is essential.

### Aetiology of cognitive problems

The aetiology of objective and subjective cognitive problems in MS is complex and not completely understood. Objective cognitive deficits in MS patients have been linked to cortical, deep grey matter and white matter damage [5, 6]. Researchers have argued that this widespread pathology may result in a disruption of the connectivity between brain regions, which in turn may result in cognitive decline [7]. Changes in brain networks are indeed present in MS patients: studies have reported changes in functional connectivity [7] and a loss of hierarchal structure [8], which both related to reduced objective cognitive performance in MS patients.

Whereas the aetiology of objective cognitive deficits is widely studied, studies focusing on the aetiology of subjective cognitive complaints are rare. Since subjective and objective cognitive problems correlate weakly [2, 3], their aetiology might be different [9]. One recent study found that subjectively experienced cognitive problems could not be explained by brain pathology, but no measures of brain networks were included [9]. To date, the study of brain networks and their relation to objective and subjective cognitive function among MS patients is still in its infancy. Additional well-designed studies are needed to unravel the aetiology of objective and subjective cognitive problems in MS.

### Treatment of cognitive problems

The best cognitive treatment options for MS patients are still a matter of debate [10]. A promising non-pharmacological treatment option is cognitive rehabilitation therapy (CRT) [10]. CRT entails the learning of new cognitive strategies aimed at compensating for cognitive problems. The use of these strategies shows positive effects on cognitive function among stroke and brain injury patients [11]. There is also some evidence for positive effects of CRT on cognitive function among MS patients. However, no final conclusion on the effectiveness of CRT can be established due to contradictory findings [12, 13]. These contradictory findings may be explained by small sample sizes, heterogeneous interventions across studies and methodological limitations (e.g. biased selection) [12, 13].

A second promising non-pharmacological treatment option is mindfulness-based cognitive therapy (MBCT) [14]. MBCT entails mindfulness training combined with elements of cognitive behavioural therapy. There is preliminary evidence that mindfulness-based interventions positively affect cognitive function in healthy individuals [14, 15] and they may even influence brain structures and functions that are involved in cognitive function [14, 16, 17]. In MS patients, positive effects of mindfulness-based interventions on psychological symptoms have been found [18–20], and a recent pilot study reported some positive effects of mindfulness on objective cognitive function [19]. To our knowledge, no other studies have investigated the effect of mindfulness-based interventions on cognitive function among MS patients. In summary, well-designed studies are necessary to investigate the effect of MBCT and CRT on cognitive function among patients with MS.

### The REMIND-MS study

The REMIND-MS study is a randomised controlled trial (RCT) that investigates the effect of CRT and MBCT on subjective and objective cognitive function in MS patients. Additionally, resting-state magnetoencephalography (MEG) data will be obtained to gain additional knowledge about the aetiology of subjective and objective cognitive problems with respect to functional brain networks, and to unravel if cognitive improvements after both interventions are associated with functional brain network changes.

### Objectives

This study primarily aims to examine the effectiveness of CRT and MBCT on subjectively experienced cognitive problems among MS patients. We hypothesise that both CRT and MBCT positively affect subjective cognitive function compared to enhanced treatment as usual (ETAU). We also expect positive effects on the secondary outcome measures objective cognitive function, functional brain network measures, psychological symptoms, well-being, quality of life and daily life functioning. Additionally, we will evaluate in an exploratory way whether there are differences in intervention effects between CRT and MBCT.

Secondary study objectives are:

1. to explore the role of functional brain network measures (using MEG) in subjective and objective cognitive problems, and to evaluate whether there are differences in functional brain network measures between these types of cognitive problems;
2. to explore the role of functional brain network measures as possible mediators in the effect of the interventions;
3. to evaluate whether alterations in objective cognitive function, functional brain network measures, psychological symptoms, well-being, quality of life and daily life functioning are mediating factors that determine subjective cognitive function;
4. to evaluate which factors determine whether a patient is likely to benefit from one of the therapies, such disease severity, severity of cognitive problems and mood at baseline, or gender.

## Methods-design

### Design

**Fig. 1:**
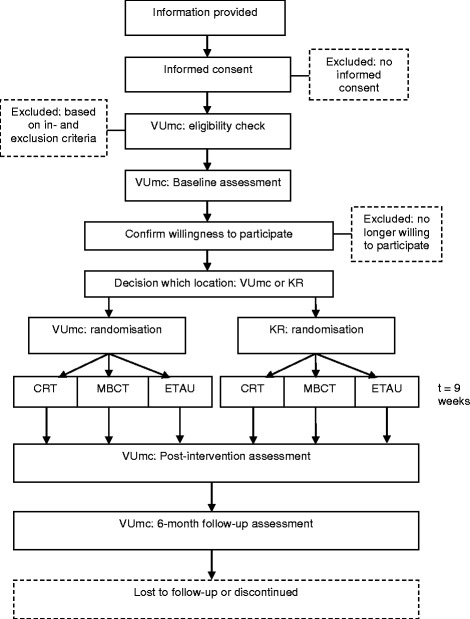
Flowchart of the trial design. KR = Klimmendaal Rehabilitation Center; VUmc = VU University Medical Center

The REMIND-MS study is a dual-centre, single-blind RCT with three parallel groups: CRT, MBCT and ETAU. All interventions last nine weeks in total. Measurements take place at baseline, post-intervention and after a 6-month follow-up period. The full trial design is summarised in Fig. 1.

### Setting

Selection and measurements take place at the VU University Medical Center in Amsterdam, the Netherlands. Part of the measurements, that is, the self-report questionnaires, can be completed by the participants at home. The interventions take place at two centres in the Netherlands: VU University Medical Center in Amsterdam and Klimmendaal Rehabilitation Center in Arnhem.

### Participants

#### Recruitment and consent

Participants are recruited through the participating centres (VU University Medical Center and Klimmendaal Rehabilitation Center), the ‘VUmc MS Center Amsterdam’ website and MS patient associations. All potentially eligible participants who express interest in the study are provided with written trial information, which contains information about the rationale, purpose and personal implications of the study. The information sheet also includes contact details of the trial coordinator and of an independent medical doctor who is not part of the research team, who can both be contacted for additional information. After sufficient time for consideration, potential participants who are still interested to participate are invited by the trial coordinator to sign the informed consent form. After signing the informed consent form, it will be checked whether the participants are fully eligible.

On the informed consent form, participants have the option to give permission for an informant to be contacted. If permission is given, an informant of the participants will also receive written information and an informed consent form, as informants will be asked to complete one questionnaire at three time-points (see outcome measures). On the informed consent form, participants and their informants also have the option to give permission for using their data for other research, for sharing their data with researchers outside of the European Union and to be contacted again for follow-up research.

#### Inclusion criteria

Participants are eligible to participate if they meet the following criteria: (1) between 18 and 65 years of age, (2) confirmed diagnosis of MS according to the McDonald 2010 criteria [21], (3) a minimum score of 23 on the Multiple Sclerosis Neuropsychological Questionnaire – Patient version (MSNQ-P), which measures subjective cognitive complaints [22].

#### Exclusion criteria

Participants who meet any of the following criteria are excluded from participation: (1) psychosis, (2) suicidal ideation, (3) an inability to speak or read Dutch, (4) previous experience with a similar intervention (e.g. a comparable cognitive rehabilitation training or mindfulness training), (5) physical or cognitive disabilities, comorbidities or treatments that would interfere too much with the interventions to enrol in this study (to be evaluated on an individual level). The reasons for excluding participants who express interest in the study will be accurately documented.

#### Sample size calculation

Mixed model analyses will be applied with three measurements comparing two groups (MBCT vs. ETAU, CRT vs. ETAU). There are no previous studies with MS patients that investigated the effect of CRT or MBCT on the primary outcome measure, the Cognitive Failure Questionnaire (CFQ) [23]. Based on a previous RCT using a comparable outcome measure, a medium effect size can be expected [24, 25]. With an alpha of .05, a power of .80, an intra-class correlation of .06, and 33 participants per group, a minimal difference of 0.62 SD can be detected between two groups. Taking into account drop-out and loss to follow-up, we intend to recruit 40 MS patients per group.

### Interventions

All interventions last nine weeks. The CRT consists of nine 2.5-h group sessions, MBCT of eight 2.5-h group sessions and one ‘silent day’, and enhanced treatment as usual (ETAU) of one individual appointment within the 9-week period. For CRT and MBCT, the optimal group size was determined based on previous experiences. Groups will consist of a maximum of 6 people in the CRT group and a maximum of 10 people in the MBCT group. Professionally trained psychologists will guide the CRT sessions, certified mindfulness trainers will teach the MBCT sessions and MS specialist nurses will have appointments with participants from the ETAU group. All trainers will be instructed and supervised by the same specialists. The interventions will be provided in a standardised manner using a written protocol. The trainers are instructed not to disclose treatment information to trainers from another treatment arm. All participants will receive an information brochure on MS and cognition.

#### Cognitive rehabilitation therapy (CRT)

The CRT protocol focuses on the following cognitive domains: speed of information processing, memory, executive function and mental fatigue. Cognitive impairments will be treated by a combination of compensatory strategy training and psycho-education. The proposed strategies are based on MS-tailored variants of evidence-based treatments that have been developed in CRT research with brain-injured subjects. Treatment of problems in information processing speed will be based on ‘Time Pressure Management’ [26, 27], memory on ‘Training Memory strategies’ [28], executive function on a ‘Multifaceted Treatment for Executive Dysfunction’ [29, 30] and mental fatigue on ‘Cognitive and Graded Activity Training’ [31, 32]. These four treatments are incorporated in the protocol as described by Geusgens, Baars-Elsinga, Visser-Meily and van Heugten [33]. In addition to cognitive strategy training, CRT focuses on emotional and behavioural changes, and grief resolution. Grief resolution will be included by explaining the stages of bereavement and by discussing the loss of physical independence, mobility, cognitive ability and emotional control on self-esteem and future perspective. The participants will receive homework assignments aimed at identifying their own cognitive problems and at applying the learned strategies in daily life situation. These homework assignments will take 30 to 45 min a day, 6 days per week.

#### Mindfulness based cognitive therapy (MBCT)

The MBCT protocol is primarily based on the MBCT program by Segal, Williams and Teasdale [34]. MBCT is an intervention in which aspects of mindfulness meditation are combined with aspects of cognitive behavioural therapy. MBCT focuses on increasing awareness of the present moment. To achieve this, participants will be trained in both self-regulation of attention and non-judgmental awareness of moment-to-moment experience. Patients will become more aware of their emotions, thoughts and behaviours and will learn to use more adaptive behaviour to respond to their symptoms. The program will be adapted to the MS patients in terms of tailoring psycho-educative elements to themes relevant to the MS patient (e.g. cognitive problems) and modified movement exercises (for patients suffering from physical impairments). Participants will receive guided mindfulness meditation exercises of 30 to 45 min, 6 days per week, for home practice and a reader with home practice instructions and background information. All therapists will fulfil the advanced criteria of the Association of Mindfulness Based Teachers in the Netherlands and Flanders, which are in concordance with those of the UK Mindfulness-Based Teacher Trainer Network [35].

#### Enhanced treatment as usual (ETAU)

Enhanced treatment as usual (ETAU) entails an appointment with an MS specialist nurse in addition to usual care. The appointment will focus on psycho-education. More specifically, the MS specialist nurse will provide the participants with information on the frequently affected cognitive domains in MS and their relation to brain pathology. This will occur in a standardised manner.

#### Teacher ratings

CRT and MBCT sessions will be recorded on video to evaluate teacher competence and protocol adherence. These video recordings will solely be used for the purpose of trainer evaluation, and the camera will be directed at the trainer. For the CRT sessions, adherence to the protocol will be checked using a checklist. For the MBCT sessions, the Mindfulness-Based Interventions - Teachers Assessment Criteria [36] will be used.

#### Adherence

For all groups, attendance to the sessions will be documented. For the CRT group, adherence to homework assignments will be checked and evaluated during each visit of the treatment period. For the MBCT group, adherence will be assessed during the entire treatment period with a calendar on which participants fill out whether they adhere to both formal (e.g. the sitting meditation) and informal (e.g. 3-min breathing space) mindfulness exercises.

#### Replacement and follow-up of withdrawn participants

Participants can leave the study at any time for any reason without any consequences. Follow-up measurements will still be scheduled if the participant is willing and able to participate in follow-up measurements. There will be no replacement of individual participants after withdrawal. If a high rate of participants drops out during the study, more participants will be included in the study. These participants will be randomly allocated to one of three parallel groups using the randomisation and minimisation procedure as described under ‘randomisation and blinding’.

#### Relevant concomitant care and interventions

During the intervention period and the 6-months follow-up period, patients are asked not to follow an intervention outside this study that focuses on mindfulness or cognition, and to keep their level of care constant during this period when possible. Naturally, usual care should continue, as do new treatment options when the health situation of the patient changes.

### Demographic and patient characteristics

At baseline, the following demographic characteristics are collected: age, gender, work status and education. In addition, the following clinical characteristics will be noted: comorbid condition as defined by the Cumulative Illness Rating Scale (CIRS) [37], subtype of MS, year of diagnosis, disease duration and MS disability as defined by the Expanded Disability Status Scale (EDSS) [38]. If an EDSS score is not available, or if this score has been determined more than three months ago, a new EDSS score will be gathered at baseline. The use of medication will be noted at each assessment. Health care consumption will be measured with a questionnaire on healthcare utilisation and productivity losses in patients with a psychiatric disorder (TIC-P) [39] and will be administered at each measurement. Table 1 presents an overview of the demographic and patient characteristics that will be collected at each assessment.

**Table 1:** Overview of outcome measures per assessment

Assessment	Baseline	Post-intervention	Follow-up
Demographic characteristics	X		
Medical history (e.g. MS subtype)	X		
Use of medication	X	X	X
Expanded Disability Status Scale (EDSS)	X		
Health care consumption	X	X	X
Questionnaires measuring subjective cognitive complaints, psychological symptoms, quality of life, well-being and daily life functioning	X	X	X
Neuropsychological assessment	X	X	X
Magnetoencephalography (MEG)	X	X	X
Qualitative data to improve the interventions		X	

### Outcome measures

All outcome measures will be administered at each assessment: at baseline, post-intervention and 6-months follow-up (see Table 1).

#### Primary outcome measure

The primary outcome measure is the level of subjective cognitive complaints and is measured with the CFQ [23]. Subjective cognitive complaints in terms of executive function will be measured with the Behaviour Rating Inventory of Executive Function – Adult Version (BRIEF-A) [40]. The BRIEF-A consists of a self- and an informant report version.

#### Secondary outcome measures

##### Cognitive function

A test battery based on the Minimal Assessment of Cognitive Function in MS (MACFIMS) will be used [41]. Verbal learning and memory is assessed with the Dutch version of the California Verbal learning Test (CVLT) [42]. Spatial learning and memory are measured with the Brief Visuospatial Memory Test-Revised (BVMT-R) [43]. Visual-spatial abilities are measured with the Benton Judgment of Line Orientation Test (JLO) [44]. Visual processing speed and working memory are measured with the Symbol Digit Modalities Test (SDMT) [45]. Verbal fluency and memory retrieval are assessed with the Controlled Oral Word Association Test (COWAT) [46]. Higher executive function is measured with the Delis-Kaplan Executive Function System sorting test (D-KEFS) free sorting condition [47]. Selective attention and response inhibition are measured with the Stroop Colour-Word Test [48]. When available, parallel versions of tests will be administered for repeated assessment to account for material-specific learning effects.

##### Functional brain networks

Resting-state MEG data will be recorded using a 306-channel whole-head MEG system (Elekta Neuromag Inc., Helsinki, Finland) in a magnetically shielded room (Vacuumschmelze GmbH, Hanau, Germany) at the VU University Medical Center. Magnetic fields will be recorded during resting state (i.e. a no-task, eyes-closed condition). Pre-processing of data and removal of noise will be done on Linux computers with available scripts [49]. The MEG data will be used to determine resting-state functional connectivity and brain network organisation. To study functional connectivity, synchronisation measures will be computed, such as the phase-lag index [7, 50]. To study brain network organisation, tools from modern network theory will be applied to the entire network and a subset of the network (i.e. the minimum spanning tree (MST)) [8, 50]. Measures such as degree, clustering coefficient and path length will be computed, as well as MST-network derived measures, such as betweenness centrality, tree hierarchy and eccentricity. There will be an emphasis on node centrality measures to identify the ‘hubs’ (i.e. highly connected nodes) of the network [51]. Since the field of modern network science is constantly developing, the best methods and measures will be selected once the study is completed.

##### Psychological symptoms

Depression and anxiety are measured with the Hospital Anxiety and Depression Scale (HADS) [52]. The level of fatigue is measured with the Checklist Individual Strength-20-r (CIS-20-r) [53]. The tendency to ruminate when being sad or depressed is measured with the subscale ‘brooding’ of the Dutch Ruminative Response Scale (RRS-NL) [54].

##### Quality of life

Health-related quality of life is measured with the Multiple Sclerosis Quality of Life Questionnaire (MSQoL-54) [55].

##### Well-being

Emotional, psychological and social well-being is measured with the Mental Health Continuum-Short Form (MHC-SF) [56]. The ability to be mindful, that is, non-judgmental awareness of moment-to-moment experience, is measured with the Five Facets of the Mindfulness Questionnaire short form (FFMQ-SF) [57]. Self-compassion, that is, the ability to act with compassion towards oneself in difficult times, is measured with the short form of the Self-Compassion Scale (SCS-SF) [58].

##### Daily life functioning

Participation in society is measured with the Utrecht Scale for Evaluation of Rehabilitation – Participation (USER-P) [59]. Goal Attainment Scaling (GAS) is used to determine the effect of the treatment on personalised goals in daily situations [60].

### Randomisation and blinding

Following baseline assessment, participants will be randomly allocated to one of three treatment arms (MBCT, CRT or ETAU). First, the location of the intervention will be determined based on the patient’s living location and preference. For each location, randomisation will be performed in variable blocks of 6 and 9, and with an 1:1:1 allocation ratio. A minimisation program will be used to ensure balance between all groups. Minimisation will be performed on three factors: (1) subjective cognitive function, (2) age and (3) gender. Weighting is equal for each factor. The minimisation program will be constructed before the start of the study by an independent scientific programmer. The randomisation procedure will be performed by a researcher who is not involved in administering any outcome measure. Outcome measurements will be administered by assessors who are blind to treatment assignment, but this blinding is not feasible with regard to participants and therapists. Prior to each post-measurement, participants will be reminded not to disclose their group allocation to the assessor.

### Data management

The collected data will be labelled with a participant identification code. The name and other identifiers of the participant will be removed from the data. The link between the participant identification code and the names of the participants will be kept separately. An electronic case report form is developed according to the guidelines of Good Clinical Practice (GCP) to document the data collected in the study. This case report form will include demographic and clinical characteristics, and all outcomes of the study parameters. The data will be treated confidentially and will only be available to the trial coordinator and principal investigator. Other investigators can only get access to the data for the purpose of research and with permission of the principal investigator. The data gathered in this study will be protected in accordance with the Dutch Personal Data Protection Act and the Medical Treatment Contracts Act.

### Statistical analysis

#### Descriptive statistics

Data on demographic and clinical characteristics will be summarised in a table. For adherence and other feasibility indicators, frequencies and percentages will be calculated. Satisfaction with the program will be summarised in qualitative descriptions. Differences between groups (CRT vs. ETAU, MBCT vs. ETAU, CRT vs. MBCT, drop-outs vs. treatment completers) in demographic and clinical characteristics and outcome measurements at baseline are analysed using independent samples t-tests (normally distributed continuous outcome variables), Mann-Whitney U tests (skewed continuous outcome variables) and Pearson’s chi-square tests (categorical outcome variables).

#### Primary and secondary objectives

To evaluate the effectiveness of the interventions, mixed-model analyses will be performed for the primary and secondary outcome measurements with time (baseline, post-intervention, follow-up) as a within subjects factor and condition (CRT vs. ETAU, MBCT vs. ETAU, and exploratory: CRT vs. MBCT) as a between-subjects factor. These analyses will be performed using an intention-to-treat approach, including all randomised participants regardless of adherence and measurement completion. Secondarily, per-protocol analyses will be performed for further exploration of the intervention effects.

To evaluate the secondary study aims, mediation and moderation analyses [61] will be performed to evaluate whether alterations in functional brain networks play a role in the effect of the interventions. In addition, cross-sectional associations between functional brain networks and cognitive function (subjective and objective) will be analysed using Pearson’s correlation and linear regression analyses. To evaluate whether alterations in secondary study parameters are mediating factors that determine subjective cognitive function, mediation analyses [61] and linear regression analyses will be performed. Finally, logistic and linear regression models will be performed to evaluate which factors determine whether a patient is likely to benefit from one of the therapies.

For all analyses, confounding variables will be inserted, such as age and education. Bonferroni corrections will be applied to correct for multiple comparisons within each objective.

### Monitoring and harms

An independent monitor, the Clinical Research Bureau (CRB) of the VU University Medical Center, will monitor the data of this study according to GCP. The CRB will check the following aspects of the participants: (1) informed consents, (2) source data verification, (3) the reported (serious) adverse events ((S)AEs). Considering the nature of this study, SAEs are not expected. All AEs that are reported spontaneously by the participant or observed by the research staff or therapists will be recorded. All SAEs will be reported by the investigator to the sponsor, and the sponsor will inform the accredited Medical Ethics Committee (MEC).

## Discussion

The best treatment options for cognitive problems in MS patients are still a matter of debate. This study will therefore investigate the effect of two promising non-pharmacological treatments: MBCT and CRT. To our knowledge, this will be the first RCT that investigates the effect of MBCT on cognitive function among MS patients. In addition, studying the effect of CRT on cognitive function may provide direction to the contradictory evidence that is currently available [12, 13]. If these treatments appear to be effective, we will investigate which factors predict this beneficial effect. These prognostic factors may lead towards tailored treatments for MS patients who suffer from cognitive problems.

An important strength of our study is that we use functional brain network measures, such as functional connectivity and nodal centrality, as an outcome variable. These measures may help to explain treatment effects and may provide information on whether network deterioration can be halted. Additionally, if functional brain network measures at baseline predict treatment outcomes, network analyses can be used as a prognostic factor. We will also relate functional brain network measures to objective and subjective cognitive problems in MS, which may help to understand the overlap and distinctiveness between these types of cognitive problems.

In summary, this study may help to unravel and treat cognitive problems among MS patients.

## Abbreviations

AE: Adverse event
BRIEF-A: Behaviour Rating Inventory of Executive Function – Adult Version
BVMT-R: Brief visuospatial memory test-revised
CFQ: Cognitive failure questionnaire
CIRS: Cumulative illness rating scale
CIS-20-r: Checklist individual strength-20-r
COWAT: Controlled oral word association test
CRB: Clinical Research Bureau
CRT: Cognitive rehabilitation therapy
CVLT: California verbal learning test
CWO: Scientific Research Committee
D-KEFS: Delis-Kaplan Executive Function System sorting test
EDSS: Expanded disability status scale
ETAU: Enhanced treatment as usual
FFMQ-SF: Five facets of mindfulness questionnaire short form
GAS: Goal attainment scale
GCP: Good clinical practice
HADS: Hospital anxiety and depression scale
JLO: Judgment of line orientation test
KR: Klimmendaal Rehabilitation Center
MBCT: Mindfulness-based cognitive therapy
MEC: Medical Ethics Committee
MEG: Magnetoencephalography
MHC-SF: Mental Health Continuum-Short Form
MS: Multiple sclerosis
MSNQ-P: Multiple Sclerosis Neuropsychological Questionnaire – Patient version
MSQoL-54: Multiple sclerosis quality of life questionnaire
MST: Minimum spanning tree
RCT: Randomised controlled trial
REMIND-MS: Cognitive Rehabilitation and Mindfulness in Multiple Sclerosis
RRS-NL: Dutch Ruminative Response Scale
SAE: Serious adverse event
SCS-SF: Short form of the self-compassion scale
SDMT: Symbol digit modalities test
TIC-P: Questionnaire on healthcare utilisation and productivity losses in patients with a psychiatric disorder
USER-P: Utrecht Scale for Evaluation of Rehabilitation – Participation
VUmc: VU University Medical Center
